# A Circulating Long Noncoding RNA Panel Serves as a Diagnostic Marker for Hepatocellular Carcinoma

**DOI:** 10.1155/2020/5417598

**Published:** 2020-07-13

**Authors:** Jinlan Huang, Yansong Zheng, Xialin Xiao, Can Liu, Jinpiao Lin, Sijia Zheng, Bin Yang, Qishui Ou

**Affiliations:** ^1^Department of Laboratory Medicine, The First Affiliated Hospital of Fujian Medical University, Fuzhou, Fujian Province 350005, China; ^2^Fujian Key Laboratory of Laboratory Medicine, Fuzhou, Fujian Province 350005, China; ^3^Department of Hepatobiliary Surgery, The First Affiliated Hospital of Fujian Medical University, Fuzhou, Fujian Province 350005, China; ^4^Medical Technology and Engineering College, Fujian Medical University, Fuzhou, Fujian Province 350004, China

## Abstract

**Background:**

Circulating long noncoding RNAs (lncRNAs) have been demonstrated to serve as diagnostic biomarkers for various cancers. We aimed to elucidate the diagnostic efficacy of eight serum lncRNAs HULC, MALAT1, Linc00152, PTENP1, PTTG3P, SPRY4-IT1, UBE2CP3, and UCA1 and their combinations for the diagnosis of hepatocellular carcinoma (HCC).

**Methods:**

A total of 129 patients with HCC, 49 patients with liver cirrhosis, 27 patients with chronic hepatitis B, and 93 healthy controls were enrolled in this study. The levels of serum lncRNAs were assessed by quantitative real-time polymerase chain reaction. The correlations between serum lncRNAs and clinicopathological characteristics were further analyzed. The receiver operating characteristic (ROC) curve and area under curve (AUC) were utilized to estimate the diagnostic capacity of serum lncRNAs and their combination with AFP for HCC. A logistic regression model was performed to establish a multiple-lncRNA panel.

**Results:**

The levels of serum HULC, MALAT1, Linc00152, PTTG3P, SPRY4-IT1, UBE2CP3, and UCA1 were significantly higher in HCC patients than in patients with benign liver diseases and healthy controls, whereas serum PTENP1 was significantly decreased in HCC patients compared with healthy participants. Positive correlations between serum Linc00152 and GGT, serum PTTG3P and GGT, and serum SPRY4-IT1 and ALT were noted in HCC patients. ROC analysis revealed that all these lncRNAs had a significantly predictive value for HCC except for PTENP1. The best performance of single lncRNA was obtained by Linc00152 with an AUC of 0.877. When combined with AFP, the combination of Linc00152 and AFP gained the highest accuracy, yielding an AUC of 0.906. Through logistic regression analysis, the panel consisting of serum linc00152, UCA1, and AFP provided the greatest predictive ability, obtaining an AUC of 0.912 with 82.9% sensitivity and 88.2% specificity.

**Conclusion:**

The panel of serum Linc00152, UCA1, and AFP demonstrates a novel and noninvasive biomarker with relatively high sensitivity and specificity for HCC diagnosis.

## 1. Introduction

Hepatocellular carcinoma (HCC) is one of the most common cancers globally with relatively high morbidity and mortality [1]. Despite great progresses made in HCC therapy in recent years, the prognosis of HCC patients still remains poor due to being diagnosed at an advanced stage and high rate of recurrence [2]. Currently, histological examination of the tumor tissue serves as the standard procedure for definitive diagnosis of HCC, while imaging examinations including ultrasound and computer tomography are the common supplement procedures [3]. However, histopathology may cause hemorrhage and tissue damage, and imaging examinations are expensive and lack adequate sensitivity and specificity. For serum biomarkers, alpha fetoprotein (AFP) is one of the most commonly used biomarkers for HCC screening and diagnosis clinically [4]. Nevertheless, the sensitivity and specificity of AFP are unsatisfactory, especially for patients with early-stage HCC [5]. Therefore, it is of great value to develop novel and noninvasive biomarkers with improved sensitivity and specificity for HCC diagnosis and prognosis.

Accumulating evidence highlights the critical roles of long noncoding RNA (lncRNA) in the initiation and progression of HCC. The lncRNAs, which are defined as noncoding RNA molecules > 200 nucleotides in length, have been confirmed to play crucial roles in various biological processes involved in hepatocarcinogenesis, including cell growth, angiogenesis, and metastasis [6]. In addition, previous reports have confirmed the stable presence of lncRNAs in various body fluids such as plasma, serum, urine, gastric juice, and saliva [7]. Several circulating lncRNAs have been implied to be promising markers with high accuracies and efficiencies for the diagnosis and prognosis of HCC [8–10]. Examples include HULC, MALAT1, Linc00152, UCA1, SPRY4-IT1, and UBE2CP3 [9, 11]. However, the diagnostic value of many lncRNAs for HCC reported by different researchers remains controversial and needs further investigation. Moreover, the diagnostic efficacy of only a small number of circulating lncRNAs has been confirmed. The majority of circulating lncRNAs involved in HCC still remains largely unclear.

Previous studies have reported that serum PTENP1 can distinguish patients with gastric cancer or clear cell renal cell carcinoma from healthy controls [12, 13] and PTENP1 functions as a vital tumor suppressor for HCC [14]. Moreover, we previously found PTTG3P plays an important role in HCC progression and can be detectable in the serum of HCC patients [15]. Therefore, in this study, we initially enrolled PTENP1, PTTG3P, and other six lncRNAs (HULC, MALAT1, Linc00152, SPRY4-IT1, UBE2CP3, and UCA1) which have been already reported to be promising serum markers for HCC [9, 11, 15]. This study is aimed at evaluating the serum levels of these eight lncRNAs among patients with HCC, chronic hepatitis B (CHB), and liver cirrhosis (LC) and healthy participants and at assessing the diagnostic values of these lncRNAs and their combination with AFP, thus to identify a panel of circulating lncRNAs as a tool for the diagnosis of HCC.

## 2. Materials and Methods

### 2.1. Patients

A total of 298 individuals were enrolled in the study (129 patients with HCC, 27 patients with CHB, 49 patients with LC, and 93 healthy controls). All patients were recruited from the First Affiliated Hospital of Fujian Medical University between September 2017 and May 2019. The patients with HCC were histologically confirmed. Patients with CHB were diagnosed according to the Clinical Practice Guidelines on the management of hepatitis B virus infection of EASL in 2017. Patients with LC were verified according to the American Association for the Study of Liver Diseases Practice Guidelines. Clinical characteristics of patients were obtained from the medical records retrospectively, including age, gender, HBV surface antigen (HBsAg), tumor number, and tumor size. As a healthy control, 93 individuals who performed their annual health check at the hospital and did not have any liver diseases or other cancerous diseases were recruited. Written informed consent was obtained from each participant, and the study was approved by the Ethics Committee of the First Affiliated Hospital of Fujian Medical University (2018[048]).

### 2.2. Sample Collection

Peripheral blood samples were collected in a separate vacuum tube from all participants before surgery, pharmacological intervention, or chemotherapy. The serum was isolated by centrifugation at 3,000 rpm for 10 min. After that, the isolated serum was immediately transferred and then stored at −80°C for further analysis.

### 2.3. RNA Isolation and Quantitative Real-Time PCR (qRT-PCR)

Total RNA was extracted from serum samples using a Hipure Liquid RNA Kit (Cat# R4163-03, Magen, Guangzhou, China). The RNA quantity and purity were evaluated via the NanoDrop One spectrophotometer (Thermo Scientific, Wilmington, DE, USA). The purified RNA was reversely transcribed into cDNA using the M-MLV Reverse Transcriptase (Cat# M1701, Promega, Madison WI, USA). Then, the levels of lncRNAs were assessed by qRT-PCR using TB Green™ Premix Ex Taq (Cat# RR420A, Takara, Dalian, China) which was performed on the QuantStudio Real Time PCR system (Applied Biosystems, Foster City, CA, USA). All reactions were performed with the following conditions: 95°C for 30 seconds, 45 cycles of 95°C for 5 seconds, and 60°C for 30 seconds. The specificity of the PCR products was ensured by melting curve analysis following each reaction. The relative expression of each lncRNA was determined using the 2−*ΔΔ*Ct method with GAPDH as the endogenous control for data normalization. The primers used are listed in Table 1.

### 2.4. Clinical Chemistry Analysis and Serum AFP Detection

Serum alanine aminotransferase (ALT), aspartic transaminase (AST), gamma-glutamyl transpeptidase (GGT), total protein (TP), and albumin (ALB) were measured on the ADVIA 2400 clinical chemistry analyzer (Siemens, Germany). Serum AFP was detected on the Roche Cobas analyzer (Roche Diagnostics, Mannheim, Germany).

### 2.5. Statistical Analysis

Statistical analysis was carried out by SPSS 23.0 software (SPSS Inc., Chicago, IL, USA) and GraphPad prism 7.0 software (GraphPad Software, Inc., La Jolla, CA, USA). Specifically, the Kolmogorov-Smirnov test was performed to analyze the normal distribution of the variables. If the variables were normally distributed, they were presented as the mean ± standard deviations (SD) and analyzed by two-tailed Student's *t*-test or one-way ANOVA. Besides, for nonparametric data, the Mann-Whitney *U* test was used for comparisons between groups. Pearson's correlation coefficient test was applied to judge the correlations between two variables with normal distribution while Spearman's correlation coefficient test was used for variables with abnormal distribution. Values of the area under curve (AUC) obtained from receiver operating characteristic (ROC) curve were performed to evaluate the diagnostic performance of HULC, PTENP1, MALAT1, Linc00152, PTTG3P, UBE2CP3, SPRY4-IT1, UCA1, AFP, and their combinations. The multi-lncRNA panel was established by logistic regression analysis. A two-tailed *P* value of <0.05 was considered statistically significant.

## 3. Results

### 3.1. Patient Characteristics

A total of 298 individuals including 129 patients with HCC, 27 patients with CHB, 49 patients with LC, and 93 healthy controls were enrolled into this study.  Table 2 lists the detailed demographic information of all participants. The median age was 59 years (range, 23-88 years) for patients with HCC, 58 years (range, 35-87 years) for patients with LC, 52 years (range, 33-71 years) for patients with CHB, and 55 years (21-79 years) for healthy controls. The HCC cohort consisted of 111 males and 18 females. For patients with LC, 32 patients were male and 17 were female. The CHB group contained 21 males and 6 females. As for healthy controls, 69 participants were male and 24 were female.

### 3.2. Serum Levels of lncRNAs in Patients with HCC, LC, and CHB and Healthy Controls

**Figure 1:**
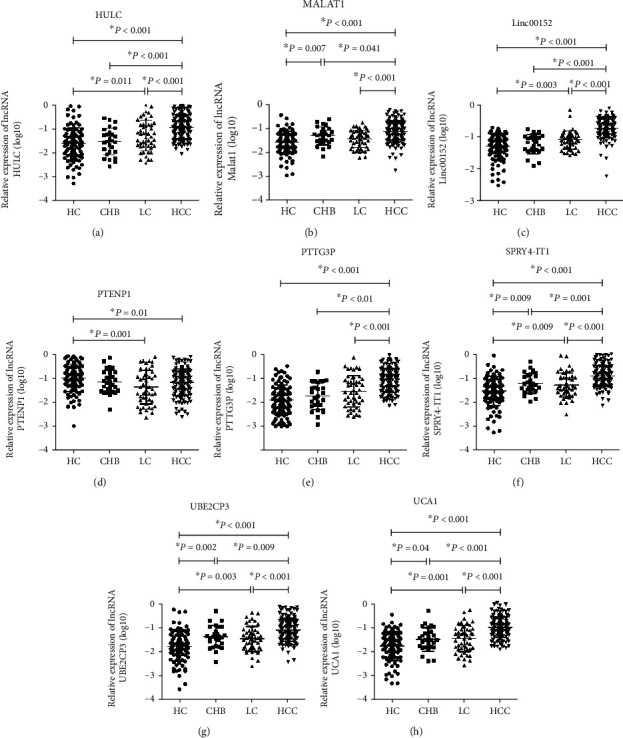
The levels of eight serum lncRNAs in patients with HCC, CHB, LC and healthy controls. The relative expression levels of lncRNAs HULC (a), MALAT1 (b), Linc00152 (c), PTENP1 (d), PTTG3P (e), SPRY4-IT1 (f), UBE2CP3 (g), and UCA1 (h) in the serum of patients with HCC (*n* = 129), CHB (*n* = 27), LC (*n* = 49), and healthy controls (*n* = 93) by qRT-PCR. Circulating lncRNA expressions were calculated using 2−*ΔΔ*Ct method. GAPDH was used as a normalization control. Data of relative expression of serum lncRNAs fitted normal distribution after log10 transformation, and thus, the independent *t*-test was applied for comparisons between groups.

As shown in Figure 1, significantly high levels of lncRNA HULC, MALAT1, Linc00152, PTTG3P, SPRY4-IT1, UBE2CP3, and UCA1 were observed in patients with HCC when compared with healthy controls, CHB patients, or LC patients. Serum PTENP1 was markedly decreased in HCC patients than in healthy controls, whereas there was no significant difference with respect to serum PTENP1 between HCC patients and CHB patients or LC patients (Figure 1(d)). Elevated levels of HULC, Linc00152, SPRY4-IT1, UBE2CP3, and UCA1 and the declined level of PTENP1 were observed in patients with LC relatively to healthy controls (Figure 1). Surprisingly, the levels of all eight lncRNAs showed no significance between the LC and CHB groups (Figure 1). Patients with CHB had higher levels of MALAT1, SPRY4-IT1, UBE2CP3, and UCA1 than healthy controls while the levels of other four lncRNAs exhibited no statistical difference (Figure 1).

### 3.3. Correlations between Serum lncRNAs and Clinicopathological Characteristics

**Figure 2:**
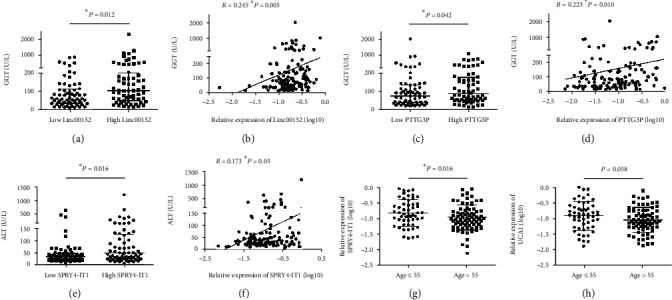
Correlation between serum lncRNAs and clinicopathological characteristics. (a) The level of GGT in patients with low level of Linc00152 (*n* = 65) was significantly lower than that in patients with high level of Linc00152 (*n* = 64). Statistical differences were analyzed by the independent *t*-test. (b) The relative expression of serum Linc00152 was positively correlated with GGT in 129 HCC patients. The linear correlation was calculated by Pearson's correlation analysis. (c) Patients with high expression of serum PTTG3P (*n* = 64) had a higher level of GGT than those with low expression of serum PTTG3P (*n* = 65). (d) Pearson's correlation analysis revealed a positive correlation between the serum levels of PTTG3P and GGT. (e) High levels of ALT were observed in patients with high SPRY4-IT1 relatively to those with low SPRY4-IT1. (f) The relative level of SPRY4-IT1 was positively correlated to ALT. Patients older than 55 years tended to have lower levels of SPRY4-IT1 (g) and UCA1 (h).

We further investigated the associations between the serum levels of eight lncRNAs and clinicopathological features in patients with HCC. Our results showed that serum Linc00152 were positively correlated with GGT (*R* = 0.245, *P* = 0.005; Table 3, Figures 2(a) and 2(b)). Positive correlations were also observed between PTTG3P and GGT (*R* = 0.225, *P* = 0.01; Table 4, Figures 2(c) and 2(d)). Furthermore, HCC patients with a lower level of serum PTENP1 tended to have a higher level of AFP (*P* = 0.064, Table 4). Serum levels of serum SPRY4-IT1 were positively associated with ALT (*R* = 0.173, *P* = 0.05; Table 4, Figures 2(e) and 2(f)). HCC patients older than 55 years tended to have a lower level of SPRY4-IT1 and UCA1 (Tables 4 and 5, Figures 2(g) and 2(h)). However, we did not observe any associations between serum lncRNA and gender, HBsAg, tumor size, tumor number, AST, TP, and ALB.

### 3.4. Diagnosis Efficacy of Each Serum lncRNA in Patients with HCC

**Figure 3:**
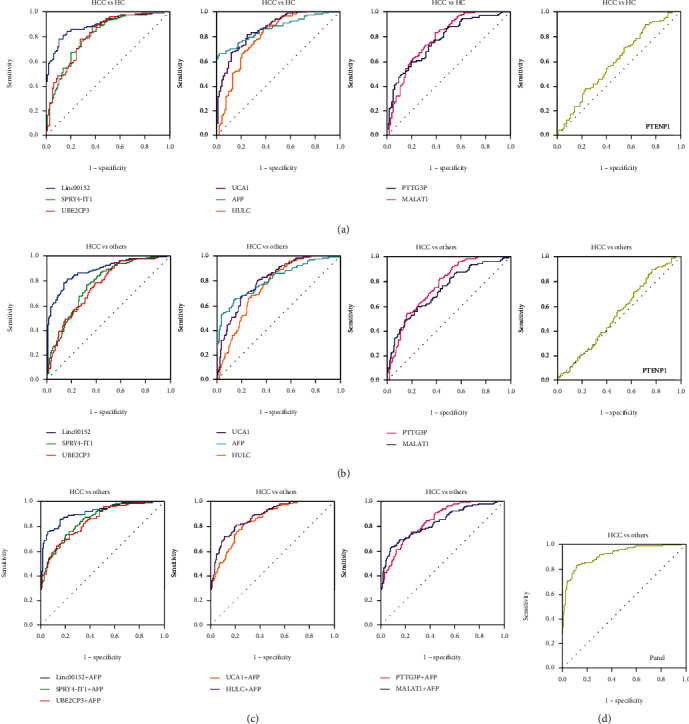
The diagnostic value of serum lncRNAs in patients with HCC. (a) The diagnostic value of HULC, MALAT1, Linc00152, PTENP1, PTTG3P, SPRY4-IT1, UBE2CP3, and UCA1 was evaluated by ROC analysis when patients with HCC were tested against patients with healthy controls. (b) Diagnosis efficacy for HULC, MALAT1, Linc00152, PTENP1, PTTG3P, SPRY4-IT1, UBE2CP3, and UCA1 to distinguish HCC patients from CHB patients, LC patients, and healthy controls. (c) Diagnosis efficacy of the combinations of serum lncRNAs with AFP to discriminate HCC from CHB patients, LC patients, and healthy controls. (d) Diagnosis efficacy of the diagnostic panel established by serum lncRNAs (Linc00512 and UCA1) and AFP.

Figures 3(a) and 3(b) show the diagnostic value (ROC curves) of 8 lncRNAs in patients with HCC. When HCC patients were tested against healthy controls, the AUC was 0.796 (0.734-0.858) for HULC, 0.768 (0.706-0.830) for MALAT1, 0.895 (0.854-0.936) for Linc00152, 0.602 (0.526-0.678) for PTENP1, 0.785 (0.723-0.847) for PTTG3P, 0.808 (0.750-0.866) for SPRY4-IT1, 0.812 (0.754-0.870) for UBE2CP3, 0.858 (0.810-0.907) for UCA1, and 0.862 (0.815-0.909) for AFP, respectively (Figure 3(a) and Table 6). Among these markers, Linc00152 demonstrated the highest accuracy in predicting HCC from healthy participants with 78.3% sensitivity and 89.2% specificity (Figure 3(a) and Table 6).

We further explored whether the relative levels of the 8 lncRNAs could discriminate HCC patients from patients with CHB and LC. As shown in Figure 3(b) and Table 6, ROC analysis showed that AFP (AUC: 0.811; 95% CI: 0.761-0.861), HULC (AUC: 0.756; 95% CI: 0.702-0.810), MALAT1 (AUC: 0.733; 95% CI: 0.676-0.790), Linc00152 (AUC: 0.877; 95% CI: 0.835-0.918), PTTG3P (AUC: 0.768; 95% CI: 0.715-0.820), SPRY4-IT1 (AUC: 0.768; 95% CI: 0.715-0.821), UBE2CP3 (AUC: 0.756; 95% CI: 0.702-0.810), and UCA1 (AUC: 0.809; 95% CI: 0.761-0.857) had a significantly predictive value for distinguishing HCC patients from CHB and LC patients and HC. However, PTENP1 (AUC: 0.530; 95% CI: 0.465-0.596) did not show any diagnosis efficacy to predicting HCC from CHB patients, LC patients, and healthy controls (*P* > 0.05, Figure 3(b) and Table 6). The best diagnostic value was obtained with Linc00152 with 81.4% sensitivity and 82.8% specificity (Figure 3(b) and Table 6).

### 3.5. Diagnosis Efficacy of the Combination of Serum lncRNAs with AFP and Establishment of Diagnostic Panel

Given the fact that AFP is one of the most common markers used for HCC screening and diagnosis, we also evaluated the combinations of these seven lncRNAs with AFP for the diagnosis of HCC. The AUC value, 95% CI, sensitivity, and specificity for the combinations of serum lncRNAs and AFP to discriminate HCC from CHB patients, LC patients, and healthy individuals are listed in Table 7. The AUC values of the combination of seven lncRNAs with AFP to predicting HCC were higher than that of AFP alone (Figure 3(c) and Table 7). The combination of Linc00152 and AFP gained the highest accuracy, yielding an AUC of 0.906 (95% CI: 0.870-0.942) with 85.3% sensitivity and 83.4% specificity (Figure 3(c) and Table 7).

Based on these data, a stepwise regression model was established to get the strongest panel for predicting HCC. Finally, two lncRNAs (Linc00152 and UCA1) and AFP were enrolled. The final formula was listed as follows: 4.528 + 3.784 × Linc00152 + 1.189 × UCA1 + 0.005 × AFP. The panel of Linc00152, UCA1, and AFP provided the greatest predictive ability, obtaining an AUC of 0.912 (95% CI: 0.878-0.945) with 82.9% sensitivity and 88.2% specificity (Figure 3(d) and Table 7).

## 4. Discussion

HCC is an aggressive tumor with high rates of recurrence and metastasis, which leads to the poor prognosis. Therefore, it is of great value to find an effective biomarker for the early diagnosis and therapy of HCC. Previous studies have confirmed that lncRNAs are detectable and also stable in serum of cancer patients, making it possible to be utilized as diagnostic biomarkers for various cancers [16, 17]. Currently, several lncRNAs have been reported to be dysregulated in the serum or plasma of HCC patients. For instance, HULC is highly expressed in tumor tissues as well as plasma of HCC patients, and plasma HULC achieves a fine diagnostic accuracy in predicting HCC [18]. However, clinical use of circulating lncRNAs for HCC still remains controversial due to inconsistent results reported by different researchers and some technical issues, such as using single or multiple lncRNAs.

In this study, we selected eight lncRNAs (HULC, MALAT1, Linc00152, PTENP1, PTTG3P, SPRY4-IT1, UBE2CP3, and UCA1) from previously published HCC-related studies [11, 14, 15, 18–20] and assessed their expressions in serum of 129 HCC patients, 49 patients with LC, 27 patients with CHB, and 93 healthy controls. Our data revealed that seven lncRNAs (HULC, MALAT1, Linc00152, PTTG3P, SPRY4-IT1, UBE2CP3, and UCA1) were markedly high expressed in serum of HCC patients compared with healthy controls, which are similar to some previous studies [11, 18, 21–23]. These results lead to an indication that these lncRNAs might be useful candidates for the screening and diagnosis of HCC. The elevation of circulating lncRNAs may result from the increased release of nucleic acids from necrosis tumor cells or increased secretion of extracellular vesicles by tumor cells into the blood [17, 24]. PTENP1 is a reported tumor suppressor for HCC [14]. Here, we revealed that serum PTENP1 was significantly downregulated in HCC patients. Moreover, MALAT1, SPRY4-IT1, UBE2CP3, and UCA1 were upregulated in CHB patients, indicating they may be involved in the early period of liver injury. The increased level of all these lncRNAs was observed in LC patients except for MALAT1. Given the fact that LC increases the risk of HCC with an annual incidence between 2 and 4% [3], these seven lncRNAs might play important roles in the early stage of HCC initiation. Intriguingly, plasma MALAT1 is reported to be upregulated in CHB patients and also LC patients [25]. In our study, significant elevation of serum MALAT1 is noted in patients with CHB while only an increasing trend is observed in LC patients. The limited sample size may be possible to be blamed. As for PTTG3P, our previous study has demonstrated the vital roles of PTTG3P in promoting cell growth and metastasis in HCC [15]. Here, we provide the first evidence that the serum level of PTTG3P is markedly high in HCC patients relatively to patients with LC and CHB and healthy controls. This finding provides a basis for the application of PTTG3P as a novel diagnostic biomarker of HCC.

Next, we also investigated the correlations between these circulating lncRNAs and clinicopathological features. A significant association between serum Linc00152 and GGT as well as serum PTTG3P and GGT was demonstrated in patients with HCC. GGT is an enzyme which can be induced by intrahepatic obstruction or directly synthesized by liver cancer cells in HCC [26]. The diagnostic value of GGT has been deeply studied, and elevated GGT has been demonstrated to predict poor survival for HCC [27]. Accordingly, serum linc00152 and PTTG3P might be associated with the prognosis for HCC patients. Our data also revealed an inverse trend between serum PTENP1 and AFP. Furthermore, we found that serum SPRY4-IT1 was positively correlated with ALT. Clinically, ALT is a useful indicator of liver injury. When liver cells are damaged, ALT will leak out from the injured cells into the blood, resulting in an increased serum ALT level [26]. Thus, it is suggested that serum SPRY4-IT1 may also be induced by liver injury. Besides, serum SPRY4-IT1 and UCA1 tended to decrease along with age in patients with HCC. In another word, younger patients tended to have higher levels of serum SPRY4-IT1 and UCA1. The same tendency of UCA1 along with age is reported by Kamel and his colleagues [28]. Some researchers found that young HCC patients have a larger tumor size and more advanced tumor stage at the time of diagnosis and thus show a worse prognosis than elder ones [29]. Therefore, we speculate that high serum levels of SPRY4-IT1 and UCA1 may serve as unfavorable prognostic factors for HCC.

To further investigate the diagnostic values of these lncRNAs, ROC curve analysis was performed. The AUC of the single lncRNA in our study is similar to some previous studies [9, 18]. We firstly investigate the diagnostic values of serum PTENP1 and PTTG3P for HCC. Serum PTTG3P was demonstrated to obtain a fine diagnostic accuracy in distinguishing HCC from LC patients, CHB patients, and healthy controls while the diagnostic potential of serum PTENP1 seems unsatisfactory. The AUC of Linc00152 was greater than those of any other circulating lncRNAs and the currently used tumor marker AFP, indicating remarkable efficacy of Linc00152 in predicting HCC from either benign liver diseases or healthy controls. Nowadays, serum AFP is the most widely used biomarker for HCC diagnosis [4]. However, its sensitivity and specificity are limited. A cohort study of 665 HCC patients carried out by Agopian et al. reported that serum AFP levels do not elevate in 31.3% of patients with HCC [30]. Therefore, we further investigate whether the combination of these lncRNAs and AFP could improve the diagnostic efficacy of single AFP. Our data revealed that the AUC of all the combinations was greater than single AFP. The best diagnostic performance was obtained in the combined use of linc00152 and AFP with an AUC of 0.906.

Previously, most of the HCC-related lncRNAs focus on single molecule when exploring the potential of lncRNA as novel biomarkers. However, the diagnostic accuracy of any single index is limited. Several studies have already developed several multiple biomarker panels to increase diagnostic accuracy, such as the lncRNA panel established by lncRNA-LET, PVT1, PANDAR, PTENP1, and linc00963 for the diagnosis of clear cell renal cell carcinoma [13] and a panel consisting of two lncRNAs (SOX2OT and ANRIL) and three tumor markers (CEA, CYFRA21-1, and SCCA) for the detection of non-small-cell lung cancer [31]. To elevate diagnostic capacity, we identified and combined three markers (linc00152, UCA1, and AFP) by a stepwise selection model, resulting in an AUC of 0.912. The diagnostic sensitivity and specificity of this panel were higher than any single markers. Interestingly, linc00152 and UCA1 have previously been linked with cancers by others. Accumulating evidence support an important role of linc00152 in glioma [32], bladder cancer [33], and HCC [34]. Linc00152 has been demonstrated to promote cell cycle progression in HCC through the miR-193a/b-3p/CCND1 axis [35]. Additionally, elevated serum linc00152 is found in patients with gastric cancer [36], non-small-cell lung cancer [37], and also HCC. The AUC for linc00152 to predicting HCC from healthy control reported by Li et al. [18] is 0.85, which is similar to our results. Similarly, frequent upregulation of UCA1 is observed in various cancers, including lung cancer, bladder cancer, gastric cancer, liver cancer, and colorectal cancer [38]. Xiao et al. [39] reported that UCA1 regulates Snail2 expression by effectively sponging miR-203 to promote HCC progression. Moreover, several studies provide strong evidence that serum UCA1 is a potential marker for HCC diagnosis and prognosis [23, 28, 40]. Consistent with our findings, Zheng et al. [23] revealed that serum UCA1 could differentiate patients with HCC from healthy participants and benign liver diseases. These studies further strengthen our findings that circulating lncRNA linc00152 and UCA1 have high potential to act as HCC biomarkers.

There are a few limitations to our study. First, we did not validate the diagnostic efficacy of the panel in another set. Second, our study was performed at a single center with relatively limited sample size. Thus, further investigations and large-scale multicenter studies are strongly recommended to fully validate this novel panel. Third, apart from AFP, AFPL3 and PIVKA-II are also common markers for HCC diagnosis. The comparisons between the diagnostic values of these serum lncRNAs and AFPL3 and PIVKA-II for HCC are also recommended for a further study.

## 5. Conclusion

In summary, we established a diagnostic panel consisting of serum lncRNA linc00152, UCA1, and tumor marker AFP with superior sensitivity and specificity to diagnose HCC. This panel might serve as a novel and noninvasive biomarker for HCC diagnosis.

## Figures and Tables

**Table 1:** Sequences of primers used in this study.

qRT-PCR primers		Sequences
HULC	Sense	ATCTGCAAGCCAGGAAGAGTC
	Antisense	CTTGCTTGATGCTTTGGTCTGT
MALAT1	Sense	AAAGCAAGGTCTCCCCACAAG
	Antisense	GGTCTGTGCTAGATCAAAAGGCA
Linc00152	Sense	GACTGGATGGTCGCTGCTTT
	Antisense	CCCAGGAACTGTGCTGTGAA
PTENP1	Sense	CATTCTTGCATGTATTTGGGTTAGG
	Antisense	GGTATATGGTCCAGAGTCCAGC
PTTG3P	Sense	GGGGTCTGGACCTTCAATCAA
	Antisense	GCTTTAGGTAAGGATGTGGGA
SPRY4-IT1	Sense	GTTTTTGCTGAGCTGGTGGTT
	Antisense	ATGGCTCCACTGGGCATATT
UBE2CP3	Sense	AAGTGGTCTGCCCTGTATGATG
	Antisense	GAGCTATCAATGTTGGGTTTGC
UCA1	Sense	TGCACCCTAGACCCGAAACT
	Antisense	CAAGTGTGACCAGGGACTGC
GAPDH	Sense	GGGAAACTGTGGCGTGAT
	Antisense	GAGTGGGTGTCGCTGTTGA

**Table 2:** Demographics of healthy controls and patient with CHB, LC, and HCC.

Features	HC	CHB	LC	HCC
Number	93	27	49	129
Age	55 (21-79)	52 (33-71)	58 (35-87)	59 (23-88)
Male/female	69/24	21/6	32/17	111/18
AFP (ng/mL)	2.22 (0.68-6.70)	2.62 (0.95-569.0)	4.03 (0.61-759.0)	38.50 (0.61-22484.0)
GGT (U/L)	16 (7-59)	36 (8-209)	64.5 (9-759)	80 (13-2000)
ALT (U/L)	18 (9-46)	28 (12-2112)	29.5 (8-1688)	37 (7-1198)
AST (U/L)	20 (14-38)	25 (18-1856)	54 (16-639)	41 (17-1474)
TP (g/L)	73.7 (65.1-81.8)	71.1 (59.1-77)	63.7 (48.6-78.2)	68.6 (43.4-84.8)
ALB (g/L)	45.5 (40.5-50.5)	45.1 (28-49.2)	28.8 (22.5-28.6)	40.1 (21.4-48.9)

HC: healthy control; HCC: hepatocellular carcinoma; CHB: chronic hepatitis B; LC: liver cirrhosis; AFP: alpha fetoprotein; GGT: gamma-glutamyl transpeptidase; ALT: alanine aminotransferase; AST: aspartic transaminase; TP: total protein; ALB: albumin.

**Table 3:** Correlation between serum level of lncRNAs HULC, MALAT1, and Linc00152 and clinicopathological characteristics in 129 HCC patients.

Features	HULC			MALAT1			Linc00152		
	Low (*n* = 65)	High (*n* = 64)	*P*	Low (*n* = 65)	High (*n* = 64)	*P*	Low (*n* = 65)	High (*n* = 64)	*P*
Gender			0.327			0.327			0.636
Male	54	57		54	57		55	56	
Female	11	7		11	7		10	8	
Age			0.333			0.333			0.801
≤55	24	29		24	29		26	27	
>55	41	35		41	35		39	37	
HBsAg			0.270			0.314			0.796
Positive	53	47		48	52		51	49	
Negative	12	17		17	12		14	15	
AFP level			0.781			0.323			0.127
≤20 ng/mL	29	27		31	25		33	41	
>20 ng/mL	36	37		34	39		32	23	
Tumor size			0.897			0.530			0.315
≤3 cm	21	20		19	22		18	23	
>3 cm	44	44		46	42		47	41	
Tumor number			0.611			0.668			0.611
Single	55	52		53	54		55	52	
Multiple	10	12		12	10		10	12	
GGT (U/L)	80 (14-2000)	77 (13-1034)	0.944	80 (13-2000)	84 (14-1034)	0.265	61 (13-697)	98 (15-2000)	0.012^∗^
ALT (U/L)	40 (7-451)	37 (12-1198)	0.944	37 (7-621)	37.5 (12-1198)	0.929	34 (7-668)	40 (12-1198)	0.378
AST (U/L)	47 (17-577)	41 (17-1474)	0.901	38 (18-577)	51 (17-1474)	0.142	36 (17-577)	48 (17-1474)	0.077
TP (g/L)	69.5 (56.0-80.6)	68.3 (43.4-84.8)	0.376	68.8 (55.3-84.8)	68.3 (43.4-84.2)	0.232	68.4 (46.8-79)	68.7 (43.4-84.8)	0.644
ALB (g/L)	40.3 (23.9-48.9)	39.3 (21.4-48.6)	0.564	40.3 (23.8-48.9)	39.2 (21.4-47.7)	0.170	40.0 (23.9-47.2)	40.2 (21.4-48.9)	0.888

^∗^ *P* < 0.05. AFP: alpha fetoprotein; GGT: gamma-glutamyl transpeptidase; ALT: alanine aminotransferase; AST: aspartic transaminase; TP: total protein; ALB: albumin.

**Table 4:** Correlation between serum level of lncRNAs PTENP1, PTTG3P, and SPRY4-IT1 and clinicopathological characteristics in 129 HCC patients.

Features	PTENP1			PTTG3P			SPRY4-IT1		
	Low (*n* = 65)	High (*n* = 64)	*P*	Low (*n* = 65)	High (*n* = 64)	*P*	Low (*n* = 65)	High (*n* = 64)	*P*
Gender			0.972			0.136			0.636
Male	56	55		53	58		55	56	
Female	9	9		12	6		10	8	
Age			0.124			0.185			0.016^∗^
≤55	31	22		23	30		20	33	
>55	34	42		42	34		45	31	
HBsAg			0.558			0.870			0.496
Positive	49	51		50	50		52	48	
Negative	16	13		15	14		13	16	
AFP level			0.064			0.089			0.858
≤20 ng/mL	23	33		33	23		28	27	
>20 ng/mL	42	31		32	41		36	37	
Tumor size			0.897			0.612			0.530
≤3 cm	21	20		22	19		19	22	
>3 cm	44	44		43	45		46	42	
Tumor number			0.968			0.329			0.329
Single	54	53		56	51		56	51	
Multiple	11	11		9	13		9	13	
GGT (U/L)	90 (14-1034)	79.5 (13-2000)	0.611	70 (13-2000)	85.5 (14-1034)	0.042^∗^	86 (13-2000)	65 (15-710)	0.517
ALT (U/L)	38 (12-1198)	35 (7-402)	0.202	33 (7-402)	42.5 (12-1198)	0.316	33.5 (12-621)	49 (7-1198)	0.016^∗^
AST (U/L)	50 (17-1474)	38 (17-577)	0.306	37 (18-577)	52 (17-1474)	0.095	36.5 (17-412)	50 (18-1474)	0.097
TP (g/L)	68.2 (43.4-84.8)	69.1 (55.5-80.6)	0.504	68.6 (55.5-80.6)	68.6 (43.4-84.8)	0.983	68.6 (46.8-80.6)	68.7 (43.4-84.8)	0.243
ALB (g/L)	39.4 (21.4-48.6)	40.5 (23.8-48.9)	0.301	40.3 (23.8-48.9)	39.5 (21.4-48.6)	0.539	39.6 (21.4-48.9)	40.2 (23.6-48.6)	0.468

^∗^ *P* < 0.05. AFP: alpha fetoprotein; GGT: gamma-glutamyl transpeptidase; ALT: alanine aminotransferase; AST: aspartic transaminase; TP: total protein; ALB: albumin.

**Table 5:** Correlation between serum level of lncRNAs UBE2CP3 and UCA1 and clinicopathological characteristics in 129 HCC patients.

Features	UBE2CP3			UCA1		
	Low (*n* = 65)	High (*n* = 64)	*P*	Low (*n* = 64)	High (*n* = 65)	*P*
Gender			0.636			0.972
Male	55	56		55	56	
Female	10	8		9	9	
Age			0.092			0.058
≤55	22	31		21	32	
>55	43	33		43	33	
HBsAg			0.796			0.558
Positive	51	49		51	49	
Negative	14	15		13	16	
AFP level			0.372			0.702
≤20 ng/mL	30	25		26	29	
>20 ng/mL	34	39		37	36	
Tumor size			0.803			0.803
≤3 cm	20	21		21	20	
>3 cm	45	43		43	45	
Tumor number			0.329			0.668
Single	56	51		54	53	
Multiple	9	13		10	12	
GGT (U/L)	67 (13-888)	83.5 (14-2000)	0.182	72.5 (13-2000)	81 (14-1034)	0.156
ALT (U/L)	36 (7-338)	38 (12-1198)	0.617	42 (7-402)	37 (12-1198)	0.871
AST (U/L)	37 (18-577)	47.5 (17-1474)	0.170	38.5 (17-577)	42 (17-1474)	0.821
TP (g/L)	69.5 (56.8-80.6)	67.8 (43.4-84.8)	0.171	69.1 (55.3-80.6)	68.4 (43.4-84.8)	0.858
ALB (g/L)	40.3 (23.9-48.9)	39.2 (21.4-48.6)	0.414	40.4 (23.8-47.2)	39.4 (21.4-48.9)	0.391

AFP: alpha fetoprotein; GGT: gamma-glutamyl transpeptidase; ALT: alanine aminotransferase; AST: aspartic transaminase; TP: total protein; ALB: albumin.

**Table 6:** Performance of AFP, HULC, MALAT1, Linc00152, PTENP1, PTTG3P, SPRY4-IT1, UBE2CP3, and UCA1 in HC and patients with HCC, CHB, and LC.

Method	HCC vs. HC			HCC vs. others^#^		
	AUC (95% CI)	SEN (%)	SPE (%)	AUC (95% CI)	SEN (%)	SPE (%)
AFP	0.862 (0.815-0.909)	65.9	97.8	0.811 (0.761-0.861)	65.1	85.8
HULC	0.796 (0.734-0.858)	86.0	62.4	0.756 (0.702-0.810)	86.0	55.6
MALAT1	0.768 (0.706-0.830)	59.7	80.6	0.733 (0.676-0.790)	59.7	75.7
Linc00152	0.895 (0.854-0.936)	78.3	89.2	0.877 (0.835-0.918)	81.4	82.8
PTENP1	0.602 (0.526-0.678)	89.1	29.0	0.530 (0.465-0.596)	89.1	23.1
PTTG3P	0.785 (0.723-0.847)	82.9	61.3	0.768 (0.715-0.820)	82.9	57.4
SPRY4-IT1	0.808 (0.750-0.866)	76.7	71.0	0.768 (0.715-0.821)	76.7	67.5
UBE2CP3	0.812 (0.754-0.870)	88.4	62.4	0.756 (0.702-0.810)	78.3	60.4
UCA1	0.858 (0.810-0.907)	81.4	75.3	0.809 (0.761-0.857)	67.4	80.5

^#^Others include HC, CHB patients, and LC patients. HCC: hepatocellular carcinoma; HC: healthy control; CHB: chronic hepatitis B; LC: liver cirrhosis; AFP: alpha fetoprotein; AUC: area under curve; CI: confidence interval; SEN: sensitivity; SPE: specificity.

**Table 7:** The performance for the combination of serum lncRNAs and AFP in HC, HCC patients, CHB patients, and LC patients.

Method	AUC (95% CI)	SEN (%)	SPE (%)
HULC+AFP	0.848 (0.806-0.890)	81.4	73.4
MALAT1+AFP	0.820 (0.771-0.870)	62.0	92.3
Linc00152+AFP	0.906 (0.870-0.942)	85.3	84.0
PTTG3P+AFP	0.837 (0.793-0.881)	69.0	81.1
SPRY4-IT1+AFP	0.847 (0.803-0.890)	72.9	79.3
UBE2CP3+AFP	0.837 (0.792-0.882)	63.6	87.0
UCA1+AFP	0.878 (0.841-0.916)	71.3	88.8
Panel^#^	0.912 (0.878-0.945)	82.9	88.2

HCC: hepatocellular carcinoma; HC: healthy control; CHB: chronic hepatitis B; LC: liver cirrhosis; AFP: alpha fetoprotein; AUC: area under curve; CI: confidence interval; SEN: sensitivity; SPE: specificity. ^#^Panel includes Linc00152, UCA1, and AFP. Backward stepwise selection was used to determine the diagnostic values of serum lncRNAs for HCC. In this study, the combination of Linc00152, PTENP1, UCA1, and AFP (3-lncRNA panel) was chosen as the strongest panel for diagnostic markers. The other lncRNAs were excluded by a stepwise procedure. The regression equation: 4.528 + 3.784 × Linc00152 + 1.189 × UCA1 + 0.005 × AFP.

## Data Availability

The data that support the findings of this study are not publicly available due to privacy and ethical restrictions but are available from the corresponding author on reasonable request.

## Abbreviations

AFP: Alpha fetoprotein
ALB: Albumin
ALT: Alanine aminotransferase
AST: Aspartic transaminase
AUC: Area under curve
CHB: Chronic hepatitis B
CI: Confidence interval
GGT: Gamma-glutamyl transpeptidase
HBsAg: HBV surface antigen
HC: Healthy control
HCC: Hepatocellular carcinoma
HULC: Highly upregulated in liver cancer
LC: Liver cirrhosis
lncRNA: Long noncoding RNA
MALAT1: Metastasis-associated lung adenocarcinoma transcript 1
PTENP1: Phosphatase and tensin homolog pseudogene 1
PTTG3P: Pituitary tumor-transforming 3, pseudogene
qRT-PCR: Quantitative real-time polymerase chain reaction
ROC: Receiver operating characteristic curve
SPRY4-IT1: SPRY4 intronic transcript 1
TP: Total protein
UBE2CP3: Ubiquitin conjugating enzyme E2 C pseudogene 3
UCA1: Urothelial carcinoma-associated 1.
